# Targeted images of KB cells using folate-conjugated gold nanoparticles

**DOI:** 10.1186/s11671-014-0725-y

**Published:** 2015-01-21

**Authors:** Pierson Rathinaraj, Kyubae Lee, Soo-Young Park, Inn-Kyu Kang

**Affiliations:** School of Applied Chemical Engineering, Graduate School, Kyungpook National University, 80, Daehak-ro, Buk-gu, Daegu 702-701 South Korea

**Keywords:** Folic acid, Gold nanoparticles, KB cells, Intracellular uptake

## Abstract

Mercaptosuccinic acid-coated gold (GM) nanoparticles were prepared and characterized by transmission electron microscopy and dynamic light scattering. Folic acid (F) was then conjugated to the GM to preferentially target oral squamous cancer (KB) cells with folate receptors expressed on their membranes and facilitate the transit of the nanoparticles across the cell membrane. Finally, a fluorescence dye (Atto) was conjugated to the nanoparticles to visualize their internalization into KB cells. After culture of the cells in a medium containing GM and folate-conjugated GM (GF), the interaction of surface-modified gold nanoparticles with KB cells was studied.

## Background

For more than one decade, nanometer-sized gold nanoparticles have attracted considerable attention not only because of their size- and shape-dependent optical and electronic properties that are distinctly different from the corresponding bulk materials but also due to their potential applications in thermal, catalysis, surface-enhanced Raman scattering, photoelectronic devices, biomedical diagnostics, and other related fields [1-3]. Gold nanoparticles offer important new possibilities in cancer diagnosis and therapy [4]. They can be used for the location and visualization [5-7] of tumors in their primary and potentially also secondary locations, as delivery vehicles for anticancer drugs, and in non-invasive ablation therapies. Gold nanoparticles are also novel promising biocompatible nanoprobes, exhibiting surfaces and cores with specific physicochemical properties, e.g., optical chirality [8], fluorescence [9,10], near-infrared photoluminescence, [11], and ferromagnetism [12], which provide new opportunities for clinical diagnostics. The transport of the nanoparticles to the tumor is a multistage process [13]. Systemically administered nanoparticles with tumor-binding ligands can accumulate in the tumors, owing to the more chaotic vasculature compared to non-diseased tissue [14].

As cancer remains extremely difficult to treat, effective strategies to detect it in its early stages are critical. In this respect, imaging has become an indispensable tool in clinical trials and medical practice [15]. Fluorescent bio-imaging is also of great value for visualizing the expression and activity of particular molecules, cells, and biological processes that influence the behavior of tumors and/or their responsiveness to therapeutic drugs [16]. Therefore, a wide range of fluorescent components has been explored by *in vitro* bio-imaging studies, including bio-marking of tumor tissues [9], angiogenic vasculature, and sentinel lymph nodes [17]. In this respect, several kinds of nanomaterials such as quantum dots, noble metal nanoparticles, and new hybrid nanocomposites of reduced graphene oxide and gold nanoparticles have demonstrated great potential for highly sensitive optical imaging of cancer, on both cellular and animal levels.

The multifunctional properties of nanoparticles provide unique advantages for the cancer-specific delivery of imaging and therapeutic agents [18]. Small molecules with infinitely diverse structures and properties are inexpensive to produce and have great potential as targeting moieties [19]. Folate, one of the small molecules most widely studied as a targeting moiety, is a water-soluble vitamin B6, essential for rapid cell division and growth in humans, especially during embryonic development [7,20]. Li et al. designed folate receptor-targeted hollow gold nanospheres carrying siRNA recognizing NF-κB, a transcription factor related to the expression of genes involved in tumor development [21]. Selim et al*.* demonstrated the use of folic acid-conjugated magnetites as a folate-targeted dual contrast agent for nuclear imaging [20].

In this study, we proposed the new gold nanoparticles with functions of molecular imaging and cell targeting. To produce hydrophilic and functional gold nanoparticles, mercaptosuccinic acid-coated gold (GM) nanoparticles were synthesized in an aqueous medium by the citrate reduction method at room temperature. Then, folic acid (F) was immobilized as a targeting moiety on the surface of the GM to ensure specific recognition of oral squamous cancer (KB) cells, to facilitate the uptake of nanoparticles into the cells, and to enhance the biocompatibility of the folate-conjugated GM (GF) nanoparticles. Animal fibroblast cells (NIH 3T3) were also used as a control. The surface properties of GM and GF were characterized by various physicochemical means, and the intracellular uptake of GF to the KB cells was also observed by confocal laser scanning microscope (CLSM).

## Methods

Auric chloride (HAuCl_4_), mercaptosuccinic acid (C_3_H_6_O_4_S), folic acid (C_19_H_19_N_7_O_6_), 1-ethyl-3-(3-dimethylaminopropyl)-carbodiimide (EDC), 3-(4,5-dimethylazol-2-yl)-2,5-diphenyl-2H-tetrazolium bromide (MTT), and N-hydroxy succinimide were purchased from Sigma-Aldrich Co. (St. Louis, MO, USA). Cell culture reagents, fetal bovine serum (FBS), Dulbecco's Modified Eagle Medium (DMEM, high glucose), penicillin-streptomycin, trypsin/EDTA, Dulbecco's phosphate buffer saline (PBS), Atto 680 fluorescence dye, and 4,6-diamidine-2-phenylindole dihydrochoride (DAPI, blue) cell staining kits were purchased from Gibco BRL (Carlsbad, CA, USA). NIH 3T3 cells (animal fibroblast cells) and KB cells (oral squamous cancer cells) were purchased from the Korean Cell Line Bank. All other chemicals used in this study were analytical grade and used without further purification.

### Synthesis of mono-dispersed gold nanoparticles

Gold nanoparticles were prepared according to the method reported by Anshup et al. [22]. Briefly, 100 ml of 0.05% HAuCl_4_ was added to a 250-ml round-bottom flask and boiled. Under rapid stirring, 3.5 ml of sodium citrate (1%) was added and further rapidly stirred for 15 min. After stirring for 30 min, the solution was gradually cooled to room temperature. After 15 min, the liquid was extracted and filtrated by a 0.22-mm filter paper. The prepared gold nanoparticles were dissolved and purified by centrifugation and double re-precipitation from distilled water.

### Preparation of hydrophilic gold nanoparticles

Mercaptosuccinic acid (0.16 M) was dissolved in 70 ml of water into a 250-ml three-neck round-bottom flask containing gold nanoparticles. The clear solution was stirred for 3 h under nitrogen. When the reaction was complete, water was removed by vacuum, and the residue was mixed with 4 ml of water and transferred to a centrifuge tube. Subsequently, 20 ml of water was added to the mixed solution, and the precipitated product was separated by centrifugation (3,000 rpm for 15 min) and washed with water. The prepared mercaptosuccinic acid-conjugated gold nanoparticles were dissolved and purified by centrifugation and double re-precipitation from distilled water.

### Immobilization of folic acid on the GM surface

**Figure 1:**
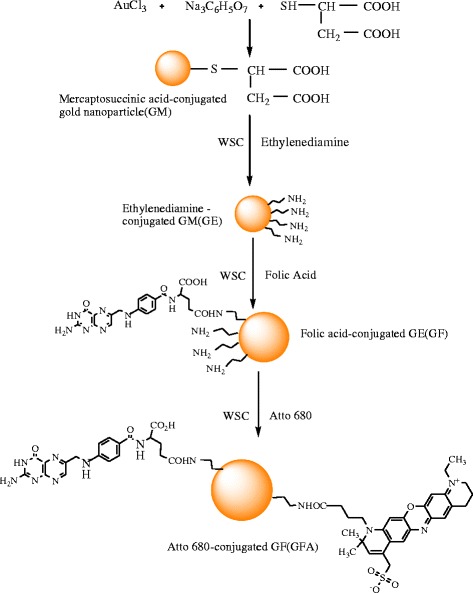
**Synthetic scheme showing the preparation of folic acid- and Atto 680-conjugated gold nanoparticles.**

Immobilization of folic acid on the GM surface involved two steps. First, GM nanoparticles were reacted with ethylenediamine to introduce primary amine groups on its surface. For this purpose, GM (0.05 g) nanoparticles were dissolved in an aqueous solution (20 ml) containing EDC (4 mg/ml) and stirred for 4 h to activate the carboxylic acid groups on the surface. Then, an excess amount of ethylenediamine was added to the solution and stirred for 24 h to obtain amine-grafted GM (GE). To keep free amine groups at one end of the ethylenediamine after the reaction, an excess amount of ethylenediamine was reacted with carboxylic acid groups on the surface. The amine-conjugated GMs were isolated via repeated centrifugation and finally dried in a freeze dryer. In the second step, folic acid was immobilized on the surface of amine-conjugated GM as follows: F (1.8 g, 0.005 mmol) was dissolved in a 0.75-N sodium citrate buffer solution (25 ml, pH 4.7) containing EDC and incubated at 4°C for 5 h to activate the carboxylic acid groups of F. Then, primary amine-conjugated GM (0.05 g) was added to the solution, and the mixture was stirred for 48 h at room temperature, to obtain F-conjugated GM, as shown in Figure 1. Finally, GF was isolated by repeated centrifugation and stored in PBS at pH 7. All the conjugation reactions, unless otherwise noted, were carried out in the dark under an N_2_ ambient environment.

### Preparation of GF conjugated to Atto dyes

GF conjugated to Atto fluorescent dye (GFA) were obtained by reacting GF (1.5 × 10^−3^ mol/l), dissolved in a carbonate buffer (pH 8), with an Atto dye (4.8 × 10^−5^ mol/l) dissolved in water at room temperature. The concentration of Atto was measured by an UV-Vis spectrophotometer (680 nm) to confirm the number of fluorophore molecules conjugated to each ligand molecule [23].

### Characterization of surface-modified gold nanoparticles

In order to confirm the presence of folic acid and Atto dye on the surface of gold nanoparticles, UV-Vis absorption spectra were recorded from aqueous dispersions at room temperature using a Hitachi U-3000 spectrophotometer (Hitachi, Tokyo, Japan). Transmission electron microscopy (TEM, Philips, Amsterdam, Netherlands; CM 200 TEM, applied operation voltage: 120 kV) was used to observe the morphology of nanoparticles. To obtain the samples for the TEM observation, the gold nanoparticles were diluted with distilled water and deposited on Formvar-coated 400 mesh copper grids (Agar Scientific, Essex, UK). After drying the nanoparticle-fluid thin film on the copper grid, a thin carbon film, approximately 10 to 30 nm in thickness, was deposited on the nanoparticles fluid film. The hydrodynamic diameter and size distribution of the gold nanoparticles were determined by dynamic light scattering (DLS) using a standard laboratory-built light scattering spectrometer with a BI90 particle sizer (Brookhaven Instruments Corp., Holtsville, NY, USA). The system was equipped with a vertically polarized incident light of 514.5 nm supplied by an argon ion laser (LEXEL Laser, model 95, Cambridge Lasers Laboratories, Inc., Fremont, CA, USA).

### Cell culture

KB and NIH-3T3 were used as target and control cells, respectively. The cells were cultured routinely at 37°C in a humidified atmosphere containing 5% CO_2_ in a polystyrene dish containing 10 ml of MEM or DMEM medium, supplemented with 10% fetal bovine serum and 1% penicillin streptomycin G sodium (PGS). The medium was changed every third day. For subculture, the cells were washed twice with PBS and incubated with a trypsin-EDTA solution (0.25% trypsin, 1 mM EDTA) for 10 min at 37°C to detach the cells. Complete media were then added to the polystyrene dish at room temperature to inhibit the effects of trypsin. The cells were washed twice by centrifugation and resuspended in complete media for reseeding and growing in new culture flasks. To observe the morphology, the cells were seeded at a concentration of 1 × 10^5^/ml in a 10-ml petri dish and incubated for 3 days with a media containing GM or GF at a concentration of 0.2 mg/ml. The morphology of the adhered cells was observed by an optical microscope (Nikon Eclipse TS100, Tokyo, Japan).

### Cell viability

To assess the cytotoxic effects of GF, after 2 days of culture, the KB and NIH-3T3 cells were suspended in PBS with a cell density of 1 × 10^5^ cells/ml. Subsequently, 200 μl of a cell suspension was mixed with a 100-μl assay solution [10 μl calcein-AM solution (1 mM in DMSO) and 5 μl propidium iodide (1.5 mM in H_2_O) mixed with 5 ml PBS] and incubated for 15 min at 37°C. The cells were then examined by fluorescence microscopy (Carl Zeiss, Axioplan 2, Jena, Germany) with 490 nm excitation for the simultaneous monitoring of viable and dead cells.

### Intracellular uptake of gold nanoparticles

To examine the cellular uptake of nanoparticles via fluorescence and confocal laser microscopy, the cells were seeded at a concentration of 1 × 10^5^/ml in a 10-ml petri dish and incubated for 1 day. The medium was then replaced with a medium containing GM and GF at a concentration of 50 μg/ml and incubated for the time (1 to 6 h) required for internalization of the nanoparticles into the cells. The cells were then washed three times with Dulbecco's PBS (D-PBS), and images were obtained using fluorescence and confocal laser microscopes. The fluorescence images were obtained using an Olympus IX70 fluorescence microscope (Olympus Corp., Tokyo, Japan) equipped with a cooled charge-coupled device (CCD) camera. The images were processed using DVC view software (version 2.2.8, DVC Company). A Zeiss LSM 410 confocal laser-scanning microscope (brightness 700 cd/mm^2^, Zeiss, Oberkochen, Germany) was used to obtain the confocal images. The position and integrity of the internalized GF nanoparticles were evaluated by confocal microscopy using 4,6-diamidine-2-phenylindole dihydrochoride (DAPI, blue) as a marker. The cell nuclei were stained by the addition of DAPI solution (10 μL) with suitable mixing and incubated for 10 min. In order to track the GF nanoparticles, F-conjugated GM and DAPI (488 nm) were added to the cells. The stained cells were washed at least three times with 1 ml of fresh DMEM medium, and images were then obtained by confocal laser microscopy [24]. To quantify the intracellular uptake of the nanoparticles, cells were grown in a 24-well culture dish with approximately 10^5^ cells in 1 ml of medium. After incubation at 37°C for 20 h, the cells were reseeded with a culture medium containing GF at a concentration of 2 × 10^4^ cell/ml. The gold nanoparticles uptaken by the cells were quantified by inductively coupled plasma (ICP). The cells were washed with PBS, detached, resuspended, counted, centrifuged down, and the cell pellets were dissolved in 37% HCl aqueous solution at 79°C to 80°C for 30 min. The samples were diluted to a final gold concentration of 1.0 to 4.0 μg/ml. The experiment was repeated three times and the results were averaged.

### Statistical analysis

The cell proliferation experiment was performed in triplicate, and the results were expressed as mean ± standard deviation (SD). The Student's *t* test was employed to assess the statistical significance of differences in the results. A difference was considered statistically significant at *p* < 0.05.

## Results and discussion

### Surface characterization

**Figure 2:**
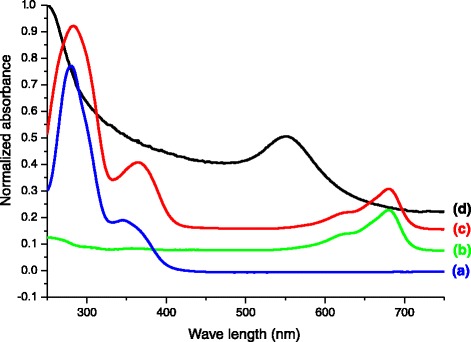
**UV-Vis spectra of surface-modified gold nanoparticles in PBS solution at pH 7.4. (a)** folic acid, **(b)** Atto 680 dye, **(c)** GFA, **(d)** GM.

**Figure 3:**
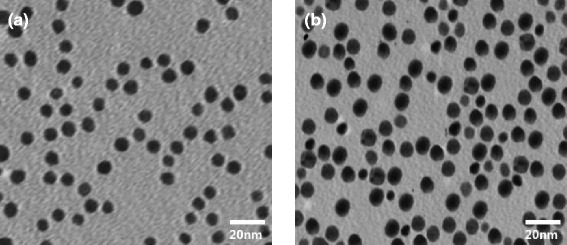
**TEM images of (a) gold nanoparticles (GM) and (b) folic acid-conjugated gold nanoparticles (GF).**

**Figure 4:**
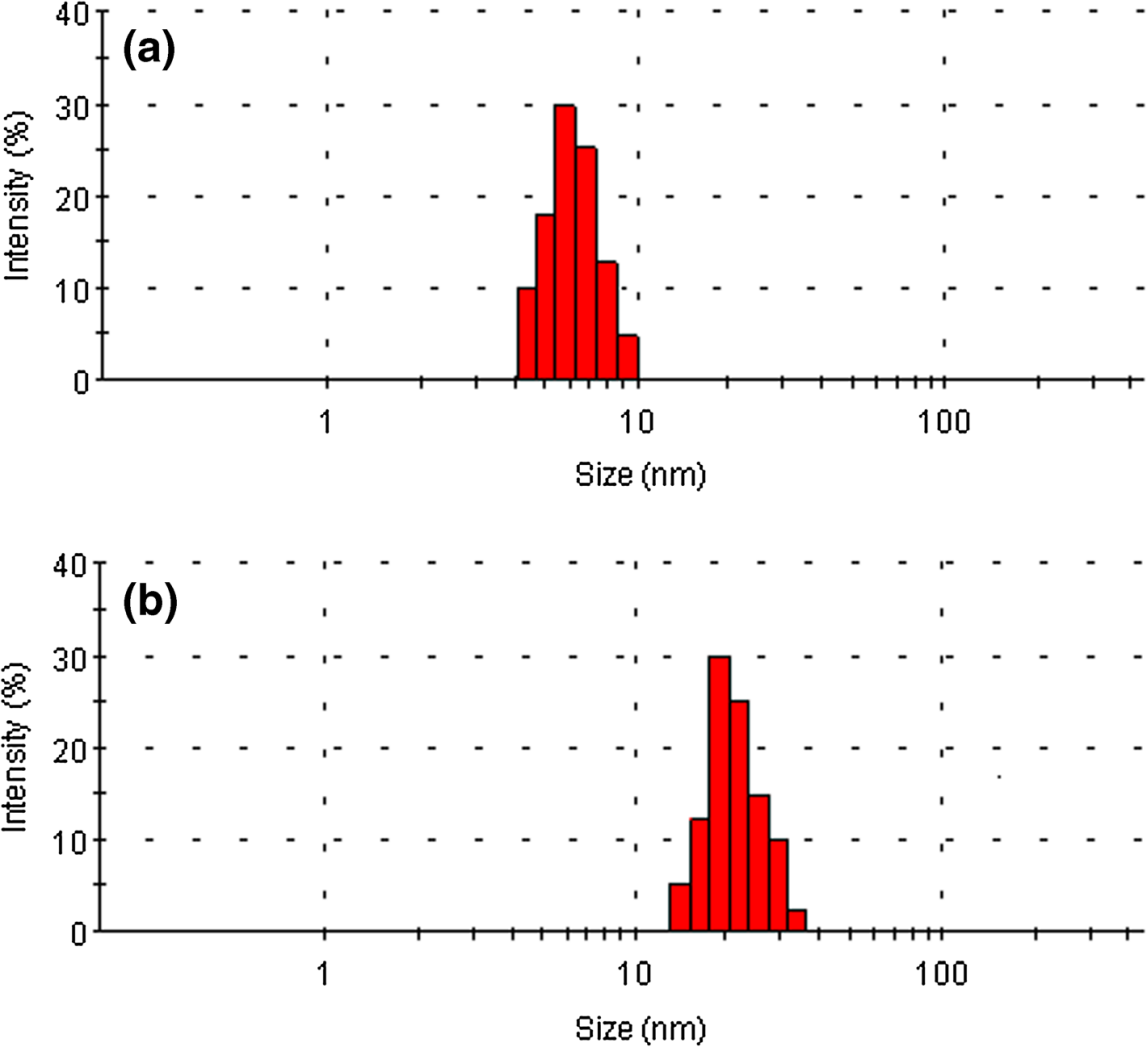
**Dynamic light scattering measurements of (a) gold nanoparticles (GM) and (b) folic acid-conjugated gold nanoparticles (GF).**

The surface modification of GM with folic acid and Atto dye was confirmed by UV-visible spectra, as shown in Figure 2. The folic acid and Atto dye exhibited a *λ*_max_ at 282 and 680 nm (Figure 2a,b), respectively. Gold nanoparticles conjugated with folic acid and Atto dye (GFA) exhibited absorptions at both 282 and 680 nm (Figure 2c), denoting the presence of folic acid and Atto dye on the gold nanoparticle surface. Figure 3a,b shows TEM images of the GM and GF nanoparticles, respectively. GM and GF have a spherical morphology with mean diameter of 6.3 and 7.1 nm, respectively. The slight increase of particle size in GF is attributed to the conjugation of folic acid on its surface. Figure 4 shows the size distribution of the synthesized GM (Figure 4a) and GF (Figure 4b) as measured by DLS. The mean diameter of GM and GF nanoparticles was 6.5 and 25 nm, respectively. The particle size, as determined by DLS, was considerably larger than that determined by TEM. This is because the DLS technique measures the mean hydrodynamic diameter of the GM core surrounded by the organic and solvation layers, and this hydrodynamic diameter is affected by the viscosity and concentration of the solution. TEM, on the other hand, measures the diameter of the core alone [25].

**Figure 5:**
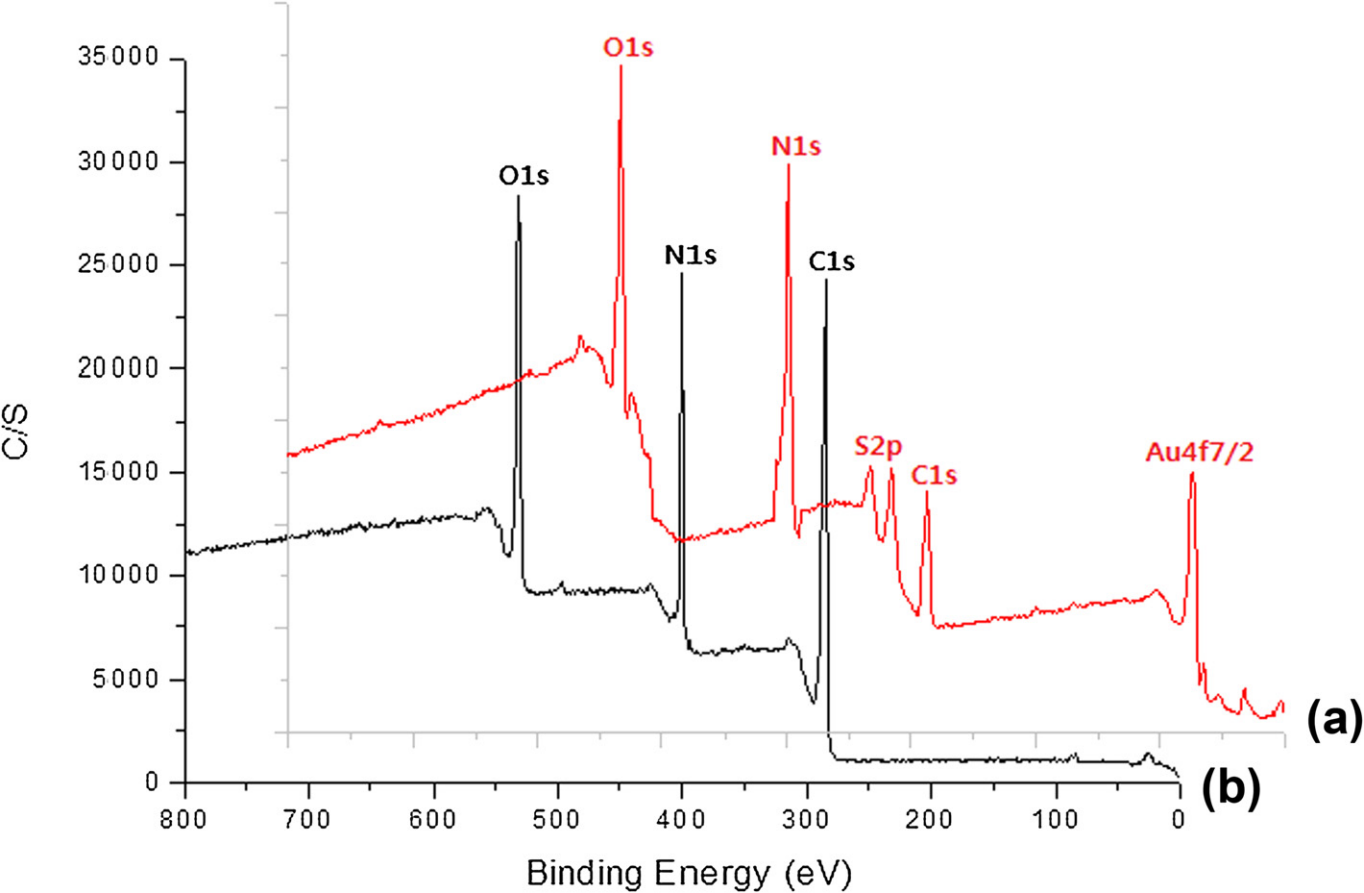
**ESCA survey scan spectra of (a) GE and (b) GF.**

The immobilization of folic acid on the surface of GE was confirmed by electron spectroscopy for chemical analysis (ESCA), as shown in Figure 5. The GE showed peaks corresponding to five elements: C 1s, O 1s, Au 4f_7_, S 2p, and N 1s (binding energies of 284.0, 526.5, 83.8, 264.05, and 397.0 eV, respectively, see Figure 5a). On the other hand, after folic acid immobilization, the GF showed the three typical peaks corresponding to C 1s, N 1s, and O 1s (Figure 5b). Table 1 lists the chemical compositions of GE and GF calculated from the ESCA survey scan spectra. The carbon and nitrogen contents of the GF (61.32% and 19.81%) were higher than that of the GE (46.0% and 15.9%). Furthermore, in case of the GF, the gold content decreased from 3.5% to 0.05% while the nitrogen one increased from 15.9% to 19.8%, indicating the successful immobilization of folic acid on the surface of GE. A possible explanation for the reduction in the Au 4f peaks is the energy loss of photoelectrons and the increase in binding energy during immobilization with folic acid [22].

**Table 1:** **Atomic percentage of GE and GF calculated from the ESCA survey scan spectra**

**Substrate**	**Atom %**				
	**C**	**O**	**N**	**S**	**Au**
GE	46.0	33.5	15.9	0.7	3.5
GF	61.32	18.41	19.81	-	0.05

### Evaluation of cytotoxicity

**Figure 6:**
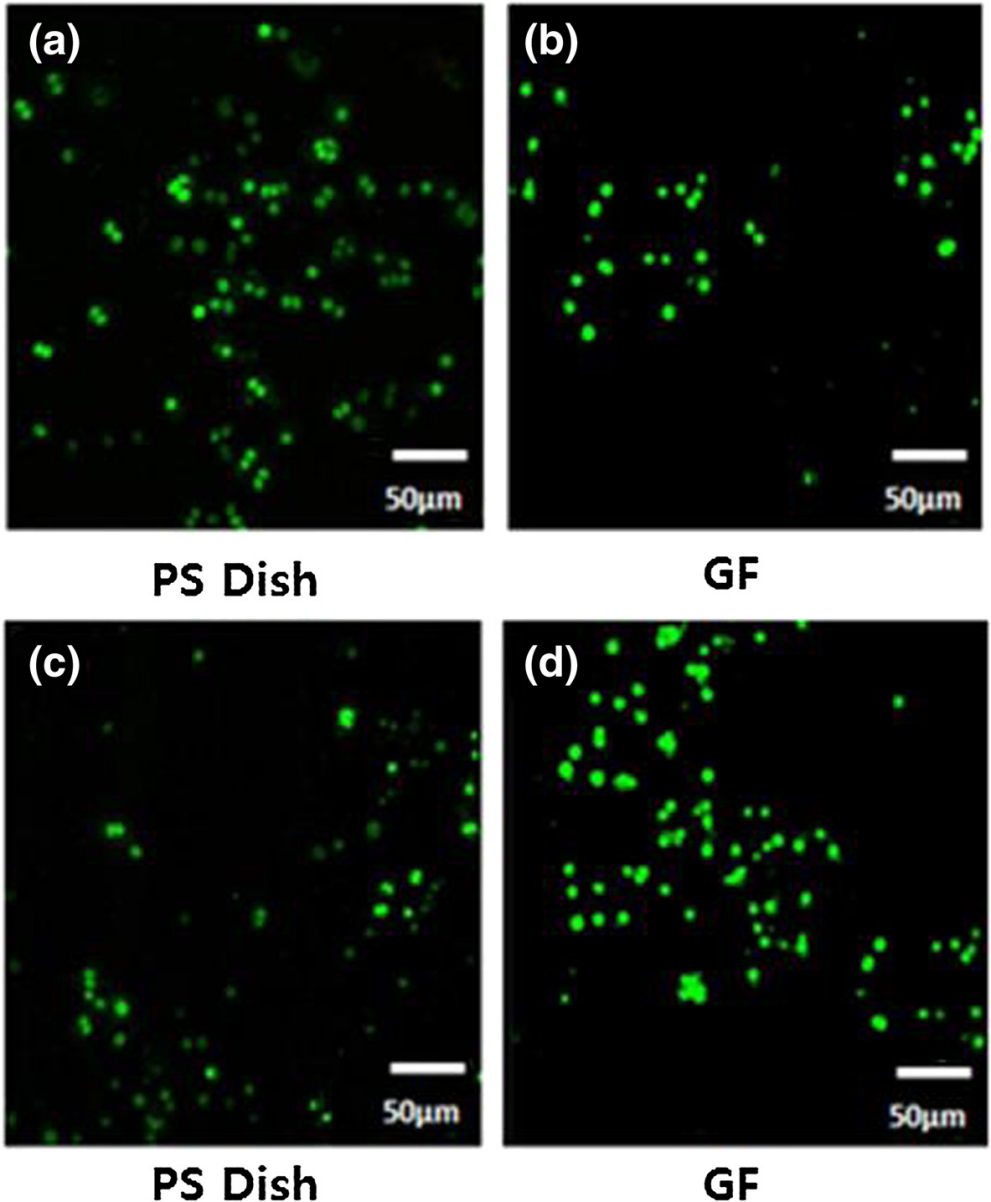
**Fluorescence microscopy images of live and dead NIH-3T3 (a,b) and KB (c,d) cells.** Images taken after culturing for 3 days in a polystyrene culture dish in the presence of culture medium **(a,c)** and GF **(b,d)**.

Figure 6 shows fluorescence microscopy images of the ‘Live/Dead’ status of dye-stained KB and NIH-3T3 cultured for 3 days in the presence of culture medium and GF. Calcein-AM (green) and propidium iodide (red) were used as markers of live and dead cells, respectively. Calcein-AM is a highly lipophilic and membrane-permeable dye. The calcein generated from the hydrolysis of calcein-AM by cytosolic esterase in a viable cell emits strong green fluorescence. Therefore, calcein-AM only stains viable cells. In contrast, propidium iodide (PI), a nuclei-staining dye, can only pass through the disordered areas of the dead cell membrane and intercalates with the DNA double helix of the cell to emit a red fluorescence (excitation: 535 nm, emission: 617 nm). After 3 days of culture, green fluorescence was observed on all GF and polystyrene culture dishes (Figure 6a,b,c,d), suggesting the presence of live cells [26]. A larger number of green fluorescence areas were identified on GF for both KB and NIH-3T3 cells, denoting good biocompatibility of folic acid-conjugated gold nanoparticles. The absence of red fluorescence in GF (Figure 6b,d) suggests that the immobilized folic acid does not have any cytotoxic effect on NIH-3T3 and KB cells, irrespective of the presence or absence of nanoparticles.

### Evaluation of intracellular uptake

**Figure 7:**
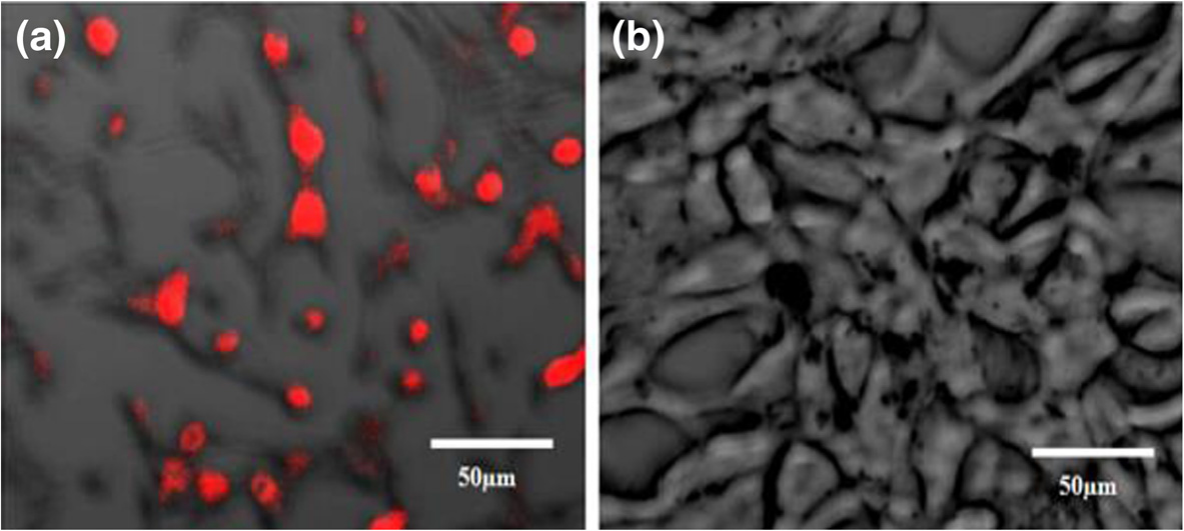
**Atto 680 dye fluorescence microscopy images of KB (a) and NIH-3T3 (b) cells.** Images show the interaction of GF with folate receptor of KB cells after culturing for 3 days in a polystyrene culture dish in the presence of culture medium and GF. The cells were stained and visualized in red under a fluorescence microscope.

**Figure 8:**
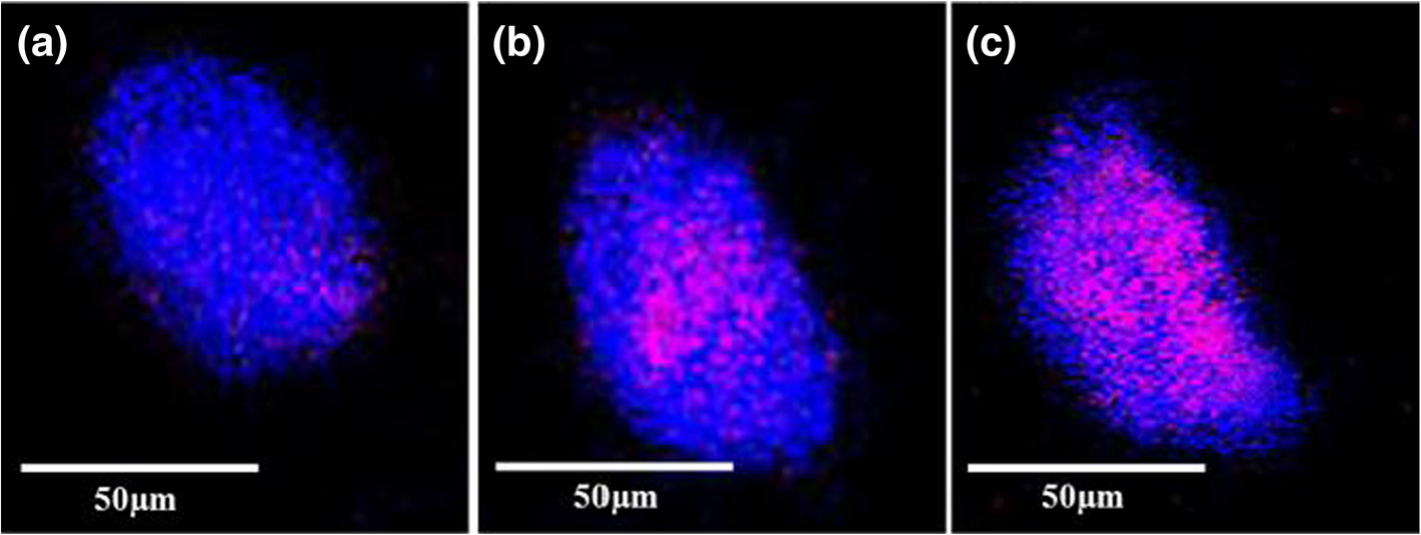
**Fluorescence images obtained from the culture of KB cells after 1, 3, and 6 h (a,b,c).** Images taken in the presence of DAPI and GF, showing specific folate receptor interaction with folic acid.

The uptake of GF into the target cells and control cells was visualized by fluorescence microscopy. Figure 7 shows fluorescence images obtained from cultured KB and NIH-3T3 cells that had been incubated for up to 6 h in the presence of GF. During cell culture in the presence of GF, a significant number of nanoparticles were transported into the KB cells and emitted intense red (Atto) fluorescence (Figure 7a). On the other hand, no significant red fluorescence was observed in NIH-3T3 cells (Figure 7b). This suggests that the GM carrying folic acid provide specific recognition signals for the nanoparticles to facilitate internalization into the target cells (KB cells). Interaction of the folic acid-conjugated GM with the folate receptors expressed on the membrane surface of the KB cells might have contributed to the improved internalization of GF into the cells, through receptor-mediated endocytosis [27]. Gan et al*.* reported similar results [28]. They introduced a hepatocarcinoma-binding peptide (A54) onto the surface of magnetite nanoparticles and examined their interaction with hepatocellular carcinoma cells *in vitro* by fluorescence microscopy. Internalization of the GF into KB occurred. Oral squamous cancer cells expressing folate receptors were quite sensitive to folic acid. Figure 7 shows that folic acid is an effective ligand, binding specifically to the folate receptor-bearing oral squamous cancer cells [29]. The internalization of GF into the KB cells was confirmed by confocal laser microscopy. Figure 8 shows the fluorescence image derived from the nucleus of the KB cells (DAPI, blue) and GF internalized (red). The cells were cultured in the presence of GF for various incubation times (Figure 8). Weak GF conjugates were observed in the fluorescence image (red color) after 1 h (Figure 8a), whereas slightly stronger fluorescence was observed after 3 h (Figure 8b), and intense fluorescence emerged after 6 h (Figure 8c). The interaction of KB cell with GF began after 1 h of incubation and was accelerated and saturated after 6 h. The confocal microscopy images suggest that the gold nanoparticle-mediated delivery of folic acid was achieved efficiently, resulting in cell internalization [30].

**Figure 9:**
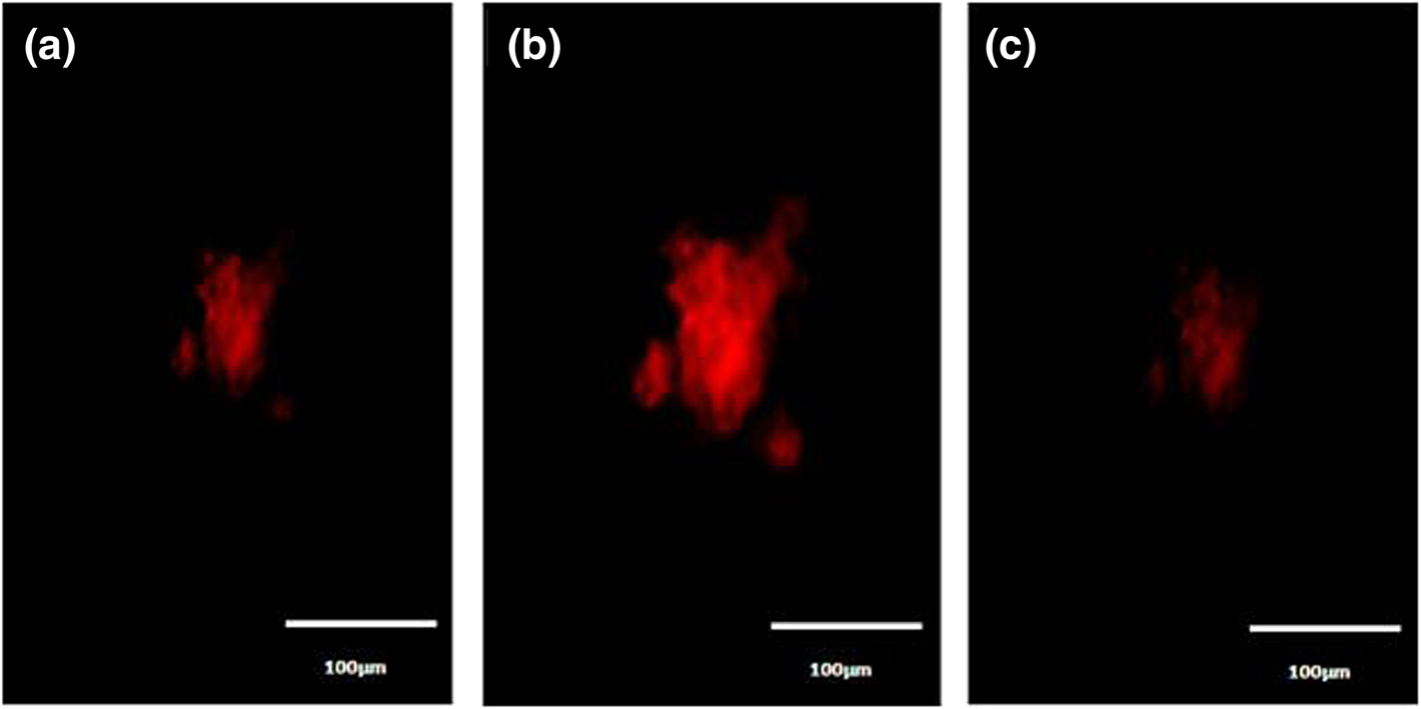
**Confocal microscopic images of KB cells cultured in the presence of GF. (a)** Top, **(b)** middle, and **(c)** bottom of the cells.

**Figure 10:**
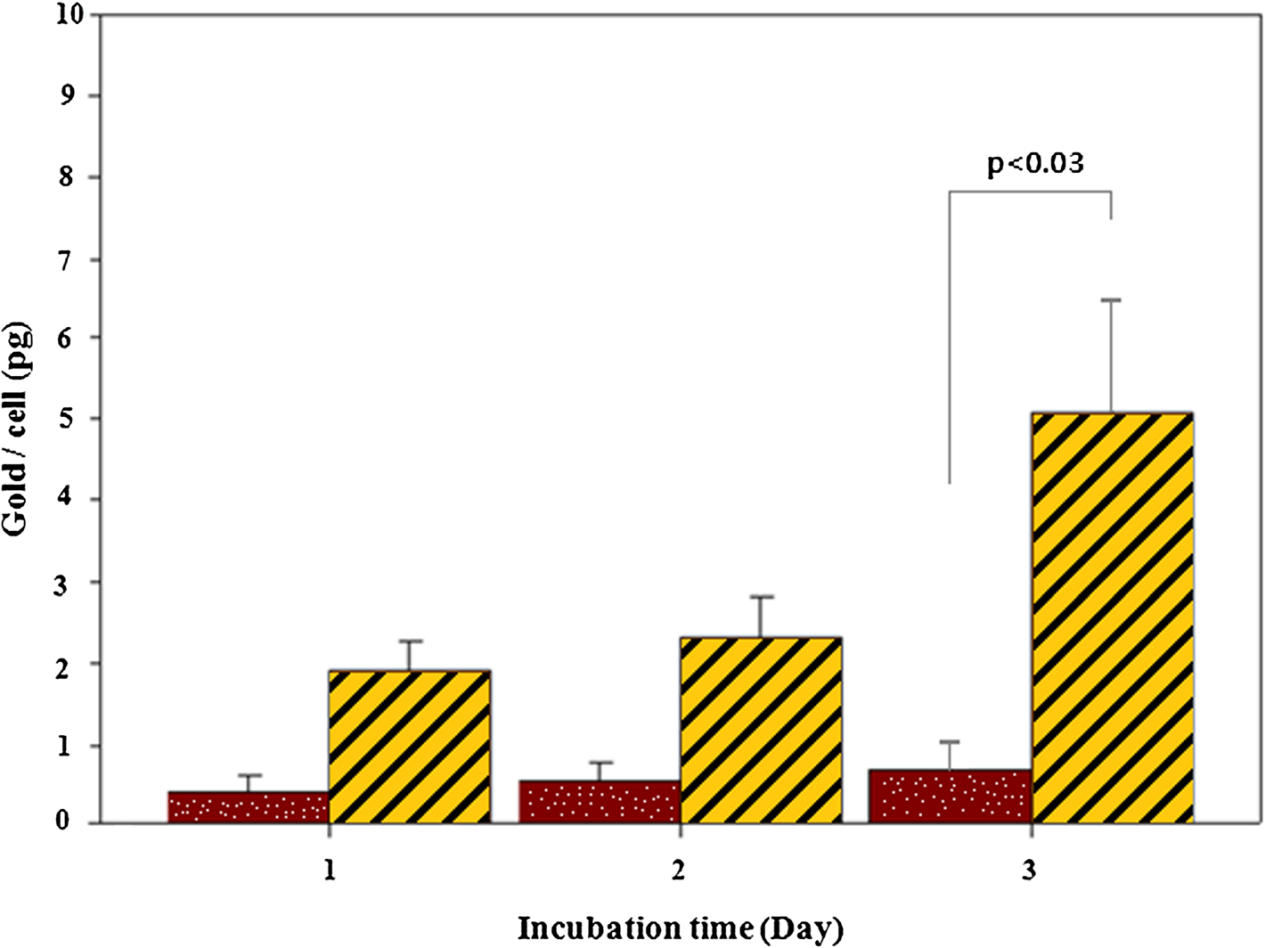
**Amount of GM (dotted red bar) and GF (yellow and black stripes bar) uptaken** **by the KB cells at different incubation time as determined by ICP (12 samples with** ***P*** **value < 0.05).**

The internalization of GF into KB cells was also confirmed by confocal microscopy performed at various depths (Figure 9), showing a weak signal at the top (Figure 9a) and bottom (Figure 9c) of the cells but intense signals in the middle (Figure 9b). This suggests that many nanoparticles are internalized in the cytoplasm, i.e., the fluorescence signal originates from the cell interior. Because the nanoparticles are rather small, receptor-mediated endocytosis is a likely mechanism for internalization [20]. The uptake of GF to KB cells was evaluated by ICP and the results are shown in Figure 10. After folic acid modification, the uptake of nanoparticles by KB cells increased greatly in comparison to modified nanoparticles and reached 1.9 pg/cell within the first day of culture, much higher than that of GM nanoparticles [31]. The maximum uptake of GF was 4.9 pg/cell after 3 days of culture. The improved uptake might be due to ligand-receptor interactions in the cell membrane [30]. Furthermore, the higher uptake of folic acid-modified gold nanoparticles (GF) indicates that the modification not only assisted the nanoparticles to target specific cells but also increased the yield of cell internalization. Similar results obtained with (carboxymethyl) chitosan-modified superparamagnetic iron oxide nanoparticles (CMCS-SPIONs) were reported by Shi et al. [32]. In their work, the amount of nanoparticles internalized in human monocyte-derived dendritic cells (hMDCs) was much more than that of SPIONs, as determined by ICP-MS. The interaction between folic acid and folate receptors expressed on the membrane of KB cells might have contributed to the improvement of GF uptake through receptor-mediated endocytosis [28].

## Conclusions

Mercaptosuccinic acid-coated gold nanoparticles were successfully conjugated with folic acid. The formation of folic acid-immobilized GM was confirmed by UV and XPS. The size of GF, as determined by DLS, was about 25 nm. The GF nanoparticles exhibited no cytotoxicity on the control (NIH-3T3) and target cells (KB). *In vitro* cell experiments showed that folic acid-immobilized GM can specifically recognize oral squamous cancer cells and emit intense fluorescence images, as well as exhibiting more efficient intracellular uptake into KB cells compared to animal skin cells (NIH3T3). These results suggest that GF has a potential for optical imaging applications and for the treatment of squamous cancer cells.
